# Role of Concomitant Internal Limiting Membrane (ILM) Peeling During Rhegmatogenous Retinal Detachment (RRD) Surgery in Preventing Postoperative Epiretinal Membrane (ERM) Formation

**DOI:** 10.12669/pjms.37.3.3576

**Published:** 2021

**Authors:** Syed Asaad Mahmood, Syed Fawad Rizvi, Burhan Abdul Majid Khan, Tanweer Hasan Khan

**Affiliations:** 1 Dr. Syed Asaad Mahmood, FCPS (Ophth) Ophthalmologist, LRBT Tertiary Eye Care Hospital, Korangi 2 ½, Karachi, Pakistan; 2 Dr. Syed Fawad Rizvi, FCPS (Ophth) Chief Consultant Ophthalmologist, LRBT Tertiary Eye Care Hospital, Korangi 2 ½, Karachi, Pakistan; 3 Dr. Burhan Abdul Majid Khan, FCPS (Ophth), FCPS (VRO) Consultant Ophthalmologist, LRBT Tertiary Eye Care Hospital, Korangi 2 ½, Karachi, Pakistan; 4 Dr. Tanweer Hasan Khan, FCPS (Ophth) Ophthalmologist, LRBT Tertiary Eye Care Hospital, Korangi 2 ½, Karachi, Pakistan

**Keywords:** Epiretinal membrane, Internal limiting membrane, Optical coherence tomography, Vitrectomy

## Abstract

**Objective:**

To investigate the role of concomitant Internal Limiting Membrane (ILM) peeling during surgery for macula off Rhegmatogenous Retinal Detachment (RRD) in preventing postoperative Epiretinal Membrane (ERM) formation; and its effect on the visual acuity.

**Methods:**

This was a prospective, quasi-experimental study conducted from August 2018 to July 2019 at LRBT Tertiary Eye Care hospital, Karachi. Fifty-six patients with macula off RRD were divided into groups A (with ILM peeling) and B (without ILM peeling) via non-probability convenience sampling. All patients underwent standard 3 ports pars plana vitrectomy with silicon oil tamponade. In Group-A, ILM was stained using 0.5% ICG. Patients were evaluated clinically and by spectral domain optical coherence tomography (SD-OCT), pre- and post-operatively. Main outcomes recorded were best corrected visual acuity (BCVA) and occurrence of ERM on SD-OCT.

**Results:**

There were 26 patients in Group-A and 30 patients in Group-B. At six months’ follow-up, ERM had not developed in any case in Group-A compared to five patients (16.7%) in Group-B. There was no statistical difference in mean BCVA change from baseline.

**Conclusion:**

ILM peeling during vitrectomy for RRD prevents the formation of macular ERM post-operatively. This may reduce the need of a second vitrectomy. However, visual outcomes were comparable to the non-ILM peeling vitrectomy.

## INTRODUCTION

Retinal detachment has come a long way from being considered a permanent cause of blindness to having up to 95% success rate with surgery.^1^ Despite the anatomical success, visual recovery tends to have a lower success ratio. Among the causes of vision impairment after rhegmatogenous retinal detachment (RRD) vitrectomy surgery, epiretinal membrane (ERM) formation on the macula remains one of the most common.^2^ These membranes were significant enough to require a further ERM removal surgery in about one third of cases.^3^

Internal limiting membrane (ILM) peeling is a technique that is routinely performed during surgery for macular diseases.^4^,^5^ Common indications for ILM peeling include various tractional vitreoretinal disorders such as macular holes, macular puckers, epiretinal membranes and diabetic macular edema. Hisatomi et al.^6^ have demonstrated the complete removal of the posterior vitreous cortex, cellular component and extracellular matrix in eyes undergoing ILM peeling.

However, despite its success in macular surgeries, the efficacy of ILM peeling in improving postoperative BCVA after vitrectomy for RRD, is still debatable.^2^ In fact, Eissa et al.^7^ found poorer visual results when ILM peeling was performed in uncomplicated RRD cases. One of the reasons for this was the thinning and reduced retinal sensitivity of the retinal area where ILM peeling was performed.^8^

The general recommendation in the studies performed on this subject over the last 10 years seemed to conclude that even though ILM peeling has been successful in reducing ERM formation in RRD patients, the visual outcomes were not favorable and this procedure might be better suited for complicated RRD cases.^2^,^9^-^13^

This study was designed keeping the results of these previous studies in view. The target patients were limited to those patients with RRD who were expected to have a higher risk of ERM formation but at the same time, the RRD was not complicated to such a degree that a poor prognosis was expected.^14^ Therefore, the results of this study would help in deciding whether concomitant ILM peeling during RRD surgery offered any benefit (in terms of ERM prevention) to patients presenting with macula-off RRD complicated with PVR formation, and whether this benefit came at a cost to the BCVA.

## METHODS

The LRBT Tertiary Eye Care Hospital ethics committee approval (Ref: LRBT/TTEH/ERC/2337/17, Dated September 27, 2017) was taken prior to the commencement of this study. This was a prospective, quasi-experimental study which included patients presenting with macula off rhegmatogenous retinal detachments. Patients were enrolled using non probability convenience sampling technique and assigned to one of the two groups. The study was conducted from August 2018 to July 2019 and the follow up duration was six months.

Complete ocular history was taken to evaluate the patient as per the inclusion and exclusion criteria. Pre-operative examination included best corrected visual acuity, intraocular pressure measurement, and fundoscopy. For assessing the extent of RRD and position of the retinal break binocular indirect ophthalmoscopy was performed using a 20D condensing lens (Volk Optical, Inc). Spectral domain optical coherence tomography (Spectralis, Heidelberg Engineering) was performed in a few patients to elucidate the presence of ERM. Written informed consent was taken from patients who fulfilled the criteria for the study, and agreed to the procedure after explanation of its benefits and risks.

### Inclusion criteria:

- Patients of primary macula-off RRD with proliferative vitreoretinopathy (PVR) grade B and C, which is not involving the macula.
- Visual acuity between 1.0 and 2.0 on logMAR chart.

### Exclusion criteria:

- Other ocular pathologies: pre-existing macular pathology (myopic maculopathy, age-related macular degeneration, macular hole), other types of RD (exudative or tractional), uveitis, retinal vascular occlusive diseases, and optic neuropathies.
- Previous history of ocular trauma and ocular surgery except for uncomplicated/uneventful phacoemulsification.
- Post-operative cataract formation significant enough to interfere with OCT imaging.
- Silicone oil removal needed before 6 months of follow-up.

### Surgical technique:

For both the groups, standardized 25-gauge pars plana vitrectomy was performed by a single vitreoretinal surgeon. Noncontact wide-angle viewing system binocular indirect ophthalmo-microscope (BIOM) and the Constellation Vision System (Alcon Laboratories, Inc.) were used. After completion of core vitrectomy, posterior vitreous detachment was induced (if not already complete) by suction of vitrectomy cutter placed closed to optic disc. Subretinal fluid (SRF) was drained through existing retinal breaks or peripheral retinotomies when necessary. The peripheral vitreous body was shaved as intensively as possible, facilitated by scleral indentation. At this stage, patients in Group-B underwent silicone oil exchange and the surgery ended.

Group-A underwent the additional procedure of ILM staining using 0.5% ICG solution (0.2 – 0.5 ml), left over the post pole for approximately two minutes and then washed away. Peeling of ILM centered at the macula was performed in circumferential fashion, aiming for a size approximately 2-disc diameters. ILM was removed by using flat tipped vitreo-retinal forceps which is used to grasp ILM, lifting it to make a break in it. Once an edge was formed, it was then removed in a circular pattern. Silicon oil was then injected at the end of the surgery.

Peeling the ILM when there is an associated retinal detachment can be a challenge, since the detached retina gives way in the direction of the peeling. Among the different techniques present for ILM peeling, the pinch and peel method worked best. Due to the lack of counter traction, flap creation with diamond dusted scraper or Finesse Flex loop (Grieshaber / Alcon) does not generally work well over detached retina. In some cases, perfluorocarbon liquid (perfluoro-n-octane, PFO) was used to provide counter-traction during the peeling process. PFO tends to flatten the elevated ILM flap towards the retinal surface so as a workaround a globule of PFO, about two disc area in size, was used to provide counter-traction allowing manipulation of the flap under balanced saline solution. This PFO globule was kept away from the leading edge of the flap by tilting the eyeball as required.

### Follow-up:

Patients were examined post-operatively on first day and then on every follow up visit which was approximately on first week, first month, third month and finally at sixth month post-operatively. On every visit, complete ocular exam was performed, which included visual acuity using the ETDRS chart, anterior segment examination, IOP assessment, posterior pole evaluation, and assessment of any complications, which were managed as required. Presence of ERM was assessed on SD-OCT at six months of follow-up.

### Statistical analysis:

IBM SPSS Statistics 25 was used to analyze the data. Frequencies with percentages were used to present qualitative variables and mean±SD were calculated for the quantitative variables. Chi-squared test was used to calculate the p-value and a value of ≤ 0.05 was considered statistically significant.

## RESULTS

Fifty-six eyes of 56 patients were included in the study. Of these, 26 patients were in Group-A and 30 patients were in Group-B. Patients’ age was 53.1±9.27 years (Group-A = 51.9±10.79 years, Group-B = 54.2±7.75 years). The difference in age was not statistically significant between the two groups (p = 0.363). Comparison of the two groups with regards to gender and eye is given in Table-I.

**Table-I:** Eye laterality and gender comparison between groups (Group-A = ILM peel, Group-B = No peel).

Peel status		No peel	ILM peel	Total	p-value
Gender	Female	14	11	25	0.477
	Male	16	1	31	
Eye	Left	13	7	20	0.159
	Right	17	19	36	

ERM formation occurred only in Group-B (non ILM peel) and this was statistically significant (Table-II). Visual acuity improved in both groups but the difference was not statistically significant at 6 months post-operative follow-up with silicone oil tamponade still in place (Table-III).

**Table-II:** Epiretinal membrane formation in the two groups.

		Peel status		Total	p-value

		ILM peel	No peel		
ERM formation	No	26	25	51	0.037
	Yes	0	5	5	
Total		26	30	56	

**Table-III:** Comparison of visual improvement in the two groups (logMAR).

		Mean	Std. Deviation	p-value
PreOp BCVA	ILM peel	1.79	0.26	0.456
	No peel	1.73	0.29	
	Total	1.76	0.27	
PostOp BCVA	ILM peel	0.75	0.32	0.874
	No peel	0.74	0.33	
	Total	0.75	0.32	
Change in BCVA	ILM peel	1.03	0.33	0.657
	No peel	0.99	0.35	
	Total	1.01	0.34	

Complications noted during postoperative visits included lenticular opacities in 5 eyes, sub-conjunctival silicone oil in two eyes and raised intraocular pressure in two eyes. There were no retinal re-detachments in any Group-At the end of six months follow-up.

## DISCUSSION

The internal limiting membrane (ILM) is the basal lamina of the inner retina which plays a crucial part in early retinal development. However, ILM tends to thicken with age and serves as a scaffold for cellular proliferation in pathologic conditions which induces tractional forces on the retina, making ILM peeling a mandatory step in the surgical management of these disorders. As epiretinal membrane (ERM) formation on the macula remains one of the most common causes of vision impairment after rhegmatogenous retinal detachment (RRD) vitrectomy surgery, ILM peeling has been attempted during primary RRD surgery for prevention of epiretinal membrane formation postoperatively.^2^

The primary aim of this study was to identify the effects of ILM peeling on ERM development in those patients with RRD who were predisposed to ERM formation (due to macular involvement and longer duration of RD) and who required a longer tamponade.^15^ Secondarily, it was assessed whether ILM peeling had any beneficial role in visual acuity improvement. The patients in the ILM peel group in this study did not develop ERM at the end of six months follow up. In contrast, a significant number of patients (16.7%) who underwent vitrectomy without ILM peeling developed ERM, six months postoperatively, as assessed by SD-OCT. However, despite the absence of ERM formation in the ILM peel group, the final BCVA achieved with the silicone oil tamponade still in place was not significantly different between the two groups.

Martinez-Castillo et al. reported an incidence of 9% of ERM formation within a year after RRD surgery.^16^ The mean BCVA had dropped to 20/63 which recovered to 20/40 after surgical removal of ERM in the same study.^16^ More recently, a meta-analysis carried out on this subject found that ERM developed in 29% of eyes undergoing RRD surgery without ILM peeling.^2^ In patients where the macula is involved by the RRD, the risk of ERM formation was shown to increase by an odds ratio of 3.81.^15^ Obata et al. reported ERM formation in 20.5% of eyes in the ILM peel group while 42.6% of eyes in the no ILM peeling group.^17^ Fallico et al. reported in a meta-analysis a retinal re-detachment rate of 3.4% in the ILM peeling group compared to 9.3% in the non-ILM peeling group, which was statistically significant in the pooled analysis.^2^ However, the studies included had used both, gas and oil, tamponade. In comparison, this study did not find any re-detachments with the use of silicon oil as tamponade.

Eissa et al. compared ERM formation and visual acuity changes in patients with uncomplicated macula-off RRD managed surgically with silicone oil tamponade, with and without ILM peeling.^7^ The study reported no ERM formation in ILM peel group vs 17% in patients in the no ILM peel group. But the improvement in visual acuity was significantly poorer in the ILM peel group. On the other hand, Chang et al. conducted a meta-analysis and reported better visual improvement in long-term follow-ups and lower ERM recurrence when ILM peeling is performed during RRD surgery.^18^ The visual gains in other studies have been mixed, with some claiming better results after ILM peeling and others reporting a worse outcome.^7^,^11^,^12^,^19^,^20^

In a relatively older study evaluating the effect of ILM peeling in macula off RRD managed with silicone oil tamponade, Aras et al. followed the two groups till three months after silicone oil removal and then compared the final visual acuity and ERM formation rates between the two groups. ERM was seen in a total of six patients in the non ILM peel group (detected in two patients before silicone oil removal and developed in four patients subsequent to silicone oil removal). However, no ERM was seen in the ILM peel group.^9^

In this study, visual acuity did improve in both groups but it was not significantly better in patients of the ILM peel group despite the lack of ERM formation in any eyes in that group. However, the short duration of the study was a major limiting factor and long-term results are yet to be seen.

An interesting observation is the lack of any correlation between the reduction in ERM formation rate with ILM peeling and the visual improvement seen across the studies. The authors suggest a few reasons, one or more of which may explain this. The use of indocyanine green (ICG) dye for staining the ILM may have influenced the visual outcomes in the ILM peel group, since dye-related toxicity has been reported in literature previously.^21^ Eyes with macula-off RRD exhibit changes in the foveal microstructures preoperatively which results in a higher incidence of photoreceptor junction disruption postoperatively thereby leading to a poorer visual recovery.^22^ The final visual acuity in this study was assessed with silicone oil tamponade in place. It is possible that with longer follow-up, or after removal of silicon oil, the occurrence and severity of ERM may increase in the non-peeling group.^17^ This, in turn, could lead the difference in the final BCVA to become significant. Similar observations were seen in a study by Bawankule et al. where a significantly higher anatomic success rate and a significantly reduced risk of redetachment was seen with ILM peeling as compared to no peel procedure at the end of a three year follow-up.^23^

## CONCLUSION

ILM peeling during vitrectomy with silicone oil tamponade for RRD prevents the formation of macular ERM post-operatively without any negative impact on visual recovery. However, the absence of ERM did not result in better visual outcome during the early post-operative period as compared to the non-ILM peeling vitrectomy group.

### Authors’ contribution:

**BAMK and SAM:** Conceptualized this study.

**SFR, SAM and THK:** Reviewed the existing relevant clinical studies and contributed to the designing of this study. Patient recruitment and initial data entry was done by.

**BAMK:** All surgeries were performed

**SAM and THK:** Statistical analysis were performed and manuscript was initially prepared.

**SFR and BAMK:** Manuscript corrected.

**SAM**: guarantor for the accuracy and integrity of this study and is the corresponding author for this study on behalf of all the authors.

All authors were involved in literature search, data acquisition and manuscript finalization.
